# Design, Analysis, and Testing of a Hybrid VTOL Tilt-Rotor UAV for Increased Endurance

**DOI:** 10.3390/s21185987

**Published:** 2021-09-07

**Authors:** Siddhant Panigrahi, Yenugu Siva Sai Krishna, Asokan Thondiyath

**Affiliations:** 1 Robotics Laboratory, Department of Engineering Design, Indian Institute of Technology Madras, Chennai 600036, India; ed18d701@smail.iitm.ac.in; 2 Department of Aerospace Engineering, Indian Institute of Technology Madras, Chennai 600036, India; ae16b115@smail.iitm.ac.in

**Keywords:** UAV, VTOL, Bi-copter, PID

## Abstract

Unmanned Aerial Vehicles (UAVs) have slowly but steadily emerged as a research and commercial hotspot because of their widespread applications. Due to their agility, compact size, and ability to integrate multiple sensors, they are mostly sought for applications that require supplementing human effort in risky and monotonous missions. Despite all of these advantages, rotorcrafts, in general, are limited by their endurance and power-intensive flight requirements, which consequently affect the time of flight and operational range. On the other hand, fixed-wing aircrafts have an extended range, as the entire thrust force is along the direction of motion and are inherently more stable but are limited by their takeoff and landing strip requirements. One of the potential solutions to increase the endurance of VTOL rotorcrafts (Vertical Take-Off and Landing Vehicles) was to exploit the thrust vectoring ability of the individual actuators in multi-rotors, which would enable take-off and hovering as a VTOL vehicle and flight as a fixed-wing aircraft. The primary aim of this paper is to lay out the overall design process of a Hybrid VTOL tilt-rotor UAV from the initial conceptual sketch to the final fabricated prototype. The novelty of the design lies in achieving thrust vectoring capabilities in a fixed-wing platform with minimum actuation and no additional control complexity. This paper presents novel bi-copter that has been designed to perform as a hybrid configuration in both VTOL and fixed wing conditions with minimum actuators in comparison to existing designs. The unified dynamic modelling along with the approximation of multiple aerodynamic coefficients by numerical simulations is also presented. The overall conceptual design, dynamic modeling, computational simulation, and experimental analysis of the novel hybrid fixed-wing bi-copter with thrust vectoring capabilities aiming to substantially increase the flight range and endurance compared to the conventional aircraft rotorcraft configurations are presented.

## 1. Introduction

Aerial flight has been a topic of sheer interest from time immemorial since the Wright brothers built the first manned aircraft in 1903. However, recent developments in lightweight composite materials, compact and reliable electronics, and robust flight controller algorithms have made the technology accessible to the research, educational, and developer communities. UAVs have created a tremendous impact on society by adding a new perspective to the way that we look at things, as they has revolutionized agriculture and farming by the application of NDVI (Normalized Difference Vegetation Index) imaging and pest control [1], remote inspection, and strategic disaster management operations through the application of image processing and object detection [2], mapping, surveillance, law enforcement, social distancing, and reconnaissance operations by providing aerial footage of the region of interest for target acquisition [3].

With the applications of UAVs catering to such widespread areas, there has been an undeniable demand for hybrid UAV configurations. UAVs have been categorically divided into three basic configurations: fixed-wing aircrafts, multi-rotors, and blimps or aerostats. Each of these configurations has advantages, limitations, and applications of their own. Yet, in recent times, research has identified various hybrid frameworks that lie at the intersection of these configurations.

One among such hybrid platforms is the VTOL fixed-wing aircraft or hybrid UAV with thrust vectoring capabilities, which could potentially address the limitations and effectively couple the advantages of both the rotorcraft and fixed-wing aircraft. The ability of having a dual flight envelope makes it ideal for complex missions where both the speed and flight endurance of the fixed-wing aircraft and hovering, aggressive maneuvering, and vertical takeoff/landing capabilities of a multi-rotor are needed [4].

These hybrid subclasses of UAVs have been studied and analyzed in the past few years due to their enormous utility [5,6]. They are further classified based on their transition mechanism between two independent flight profiles enabling them to have a dual flight envelope. We used such studies as a backbone to come up with the conceptual sketch for our prototype design. The hybrid platform of tilt-rotor UAVs has been primarily classified into two main categories as follows:

(1) Tail-sitters: This configuration takes off and lands vertically on its tail and undergoes the transition from vertical to horizontal level flight mode by differential thrust or actuation by control planes. Due to their ability to achieve level flight without any extra actuators, they turn out to be mechanically simple but pose a complex problem in control and structural design, as they need to withstand periodic impact loads on their rear tails, which is undesirable for a miniature UAV, as in our case [7].
(2) Convertiplanes: Unlike the tail sitters, convertiplanes maintain their aircraft configuration integrity throughout the flight envelope (i.e., the fuselage is level in both flight modes) and employs the tilting of the subcomponents such as a morphable wing, motor orientation, etc., to achieve seamless transfer between independent flight modes [8]. Depending on the subcomponent responsible for the transition, convertiplanes are again subcategorized into:
  (A) Dual System Tilt-Rotors: This configuration utilizes multiple actuators, which are directed upwards and downwards in a pusher tractor configuration [9]. However, this subsystem is easy to design and model, as both flight modes can be analyzed separately. However, carrying extra actuators can reduce overall efficiency due to the decreased payload carrying capacity and an increase in propeller downwash. One of the main reasons for prototyping such a hybrid VTOL vehicle was to increase the endurance, and carrying additional actuators posed a design penalty to such an objective.
  (B) Rotor wing/Stop rotor: Another bio-inspired design of the hybrid convertiplane is the rotor-wing, where the rotating wing generates lift for hovering, and after gaining a particular altitude, it decelerates and subsequently stops its motion to act similar to a fixed-wing aircraft to cruise larger distances hence the name stop-rotor. This design is best optimized in terms of structural efficiency as it ensures the complete utilization of the wing, which is one of the most significant components in terms of weight and size in any fixed-wing UAV. Nevertheless, this idea of reducing the number of redundant components is essentially a tradeoff, as it has demanding design requirements. One of these challenges or constraints is that the wing needs to have an elliptic profile for maximum lift distribution. It is required to be symmetrical to balance out the aerodynamic forces and moments [10]. The transition from a fully rotating wing in the VTOL phase to an inactive fixed-wing phase will create a gyroscopic moment and add to the challenges in attitude controller design. Finally, even though both of the forces required for the motion are different, i.e., rotational for the VTOL phase and translational for the fixed-wing phase, they need to be generated from the same frame, which makes the entire problem quite intricate [11]. Due to the above-mentioned challenges, the conceptual idea of designing a mono-copter rotor wing was not deemed feasible, as a de-spin platform or an active rotating tail also needed to be designed to make it stable during the transition phase, which would add to the complexity and would increase the power consumption of the entire design.
  (C) Tilt Wings: This subcategory, as the name suggests, achieves seamless transition by tilting the entirety of both wings with a motor fixed to them. As the motors remain fixed to the wings, dynamic modeling is simplified by adopting a wing fixed reference frame [12,13]. However, in hindsight, as the wing is directed vertically upwards during hovering, landing, and takeoff, the motor adds additional drag and makes the aircraft unstable due to its inherent susceptibility to crosswinds and gust disturbances.
  (D) Tilt-Rotor: The tilt-rotor concept is identical to that of tilt wings, where multiple rotors are mounted on the shaft or nacelles at the end of the wingspan. This solution shows promising potential, as actuation can be achieved with a minimum of two actuators, unlike dual system convertiplanes, leading to an increase in payload carrying capacity and a reduction in wing loading, as changing the orientation of the rotor shafts leads to the transition without affecting the structural integrity of the vehicle, unlike tail sitter UAVs, and is more robust and adaptable to crosswind condition, unlike tilt wings [5,12]. Few design concepts of VTOL tilt-rotors have been explored in the past, but the major research focus of past researchers was on the quad-copter or tri-copter design [14,15] and the conceptualization of robust control schemes for flight transition [15,16,17]. However, this paper focusses on achieving the same idea with a minimum number of actuators and on formulating mathematical modelling that will aid in robust control scheme implementation.

**Figure 1:**
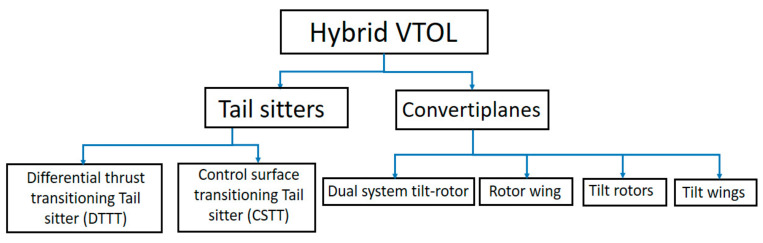
Classification of hybrid VTOL configuration.

Hence, we went forward with the VTOL tilt-rotor design with dual rotors to achieve optimal performance with minimum actuation after exploring the various design alternatives, as shown in Figure 1. Another aspect of the design was the point of actuation where the brushless DC motors are mounted on the boom, which seemed to be reasonable, as it reduces the aerodynamic interference between the propellers and wing and also ensures a uniform and smooth airstream flow over the wing. The conceptual design of the VTOL tilt-rotor with a pair of counter-rotating actuators was finalized with the objective of achieving an efficient hybrid vehicle in terms of aerodynamics and overall weight. However, this design adds to the complexity in modeling and controller design, as the entire flight profile can now be divided into three different phases, i.e., the VTOL hover phase, the transition phase, and the horizontal fixed-wing flight phase. Not only do the flight phases in the entire flight profile change, but the functionality of the control surfaces and the actuators also vary in each of these independent flight profiles. Considering an example of the axisymmetric differential thrust of the actuators, differential thrust in the hover phase leads to the roll motion of the fuselage axis of the vehicle. However, in the horizontal fixed-wing flight phase, differential thrust produces a yawing motion at the vertical axis. Hence, these challenges in design, modeling, control, simulation, and fabrication will be addressed sequentially in this paper.

This paper discusses the conceptual design and fabrication of a novel hybrid VTOL tilt-rotor to address the endurance limitation in rotorcrafts and the landing and takeoff strip requirements in fixed-wing aircrafts. The objective of the design is to address the challenge of achieving optimal performance using minimum actuation and by achieving flight stability by implementing control in all independent flight modes. This concept of thrust vectoring with dual actuators can also be implemented as a fail-safe mode in multi-copters where a pair of counter-rotating propellers can lead to a safe landing in case of failure without inflicting any harm to the physical environment. Similarly, the tilting ability of the actuators can also help to vary the attack angle of a hybrid fixed-wing UAV in order to achieve an optimum thrust to weight ratio for larger cruise distances.

The rest of the paper is organized as follows: Section 2 provides the mechanical design of the conceptualized hybrid UAV, and Section 3 emphasizes the principles of operation by that it is able to achieve under actuated motion in air. Kinematic and dynamic modelling of the hybrid UAV is discussed in Section 4. Finally, controller design and experimental results are discussed in Section 5 followed by the conclusion and future work.

## 2. Mechanical Design

The initial design of the hybrid VTOL tilt-rotor is conceptualized on two prior key ideas, which were identified as a design necessity, i.e., the minimum weight and the maximum efficiency in terms of aerodynamics. Hence, it is designed with two actuators that turned out to be the minimum number of support actuation points without introducing any additional complexity in the controller design. The two actuators are mounted on the wing boom with the help of custom-made rotor mounts and can be tilted independently by the actuation of high torque metal servos. Mounting actuators on the wing boom proved beneficial, as the design was symmetrical at the fuselage, with both actuators on either side of the CG (centre of gravity). The CG of the entire hybrid platform is at the midpoint of the wing boom, which simplified the design.

The fuselage is placed underneath the CG and could be used as a housing cabin to nest all of the flight avionics and electronics. The conventional tail acts similar to an extension of the fuselage with horizontal and vertical stabilizers. Both of the actuators present on either side of the wing can be operated in the vertical orientation for the VTOL flight phase and can be subsequently tilted by servos to the horizontal orientation used in the fixed-wing flight phase.

**Figure 2:**
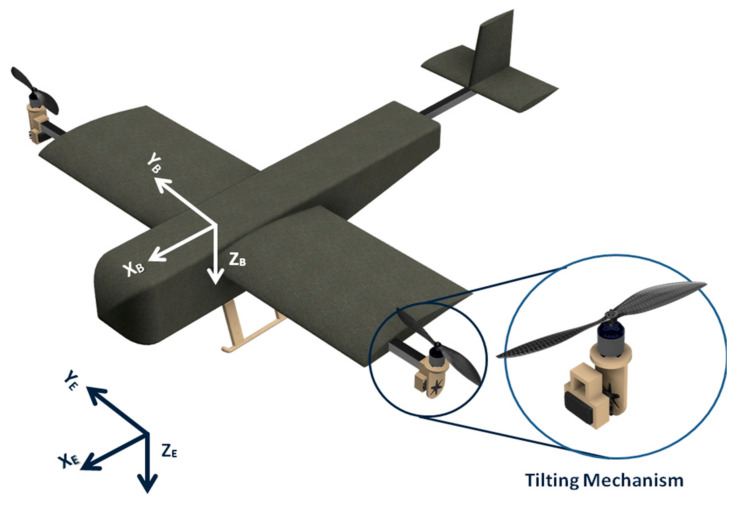
Isometric view of hybrid tilt-rotor.

Hence, the mechanical design of the hybrid VTOL tilt-rotor consists of four major parts, i.e., the wing, the fuselage, the tail, and the rotor with a tilt mechanism. An isometric schematic view of the components in the hybrid VTOL tilt-rotor along with its respective inertial (X_E_, Y_E_, Z_E_) and body (X_B_, Y_B_, Z_B_) reference frames is shown in Figure 2.

Due to rotating nacelles, the wing demanded an aerofoil cross-section to have the least amount of C_M_ (coefficient of pitching moment) variation, with a change in the attack angle preventing stalling and maximizing C_L_/C_D_ to ensure an increase in endurance and a smooth transition between two independent flight profiles. Hence, a high wing configuration with a DAE 51 aerofoil cross-section was chosen after comparing several other possible symmetrical and unsymmetrical airfoils in XFOIL [18]. Both the wing and the fuselage are supported and held together by custom-designed brackets with square aluminum rods. Custom motor mounts were also designed, where both the motor and high torque servos could be housed in a single compartment, and two coaxial spur gears would enable the tilting motion of actuators.

**Figure 3:**
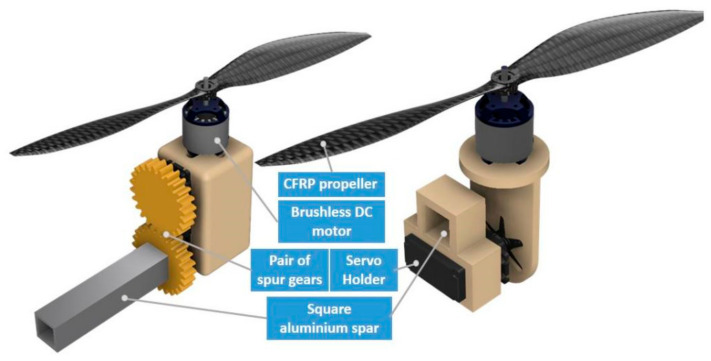
Iterations of the motor pod conceptual design.

However, there were few shortcomings in the first design that needed to be addressed. Hence a second iteration of the design was conceptualized. The first minuscule change was the change in the configuration of the tail from an H to an inverted T. This small change was due to the effectiveness of the control surface that was only present in the fixed-wing flight phase. This minor change with a slight shift in the position of the flight avionics led to a reduction in weight, as a shorter fuselage stout could now be used. The earlier design of the tilt-able rotor mount, a similar version of which was also implemented by Tom Stanton [19], was compact and elegant but had severe problems backlash problems in the spear gears. Hence, a second rotor mount was designed without the gears for the tilting mechanism, as illustrated in Figure 3.

The third and final iteration incorporated the landing gears, which were placed underneath the CG so that carrying the payload did not affect the flight dynamics. Here, both the landing gears and the payload are mounted in such a manner that the center of mass of the overall system is lying on the same axis with the center of mass of the payload and the landing gear. Hence, mounting of payload will introduce a vertical offset in the CG of the overall system along the heave axis without causing any change in the overall inertial terms. Table 1 shoes all of the critical design parameters based on the mechanical design. The overall dimensions, the center of gravity, and inertial terms were derived from the CAD design, as shown in Figure 2.

## 3. Principle of Operation

Being a hybrid framework of the multi-copter and conventional fixed-wing aircraft, the working principle of a hybrid VTOL tilt-rotor is quite similar to that of a multi-copter with slight variation in each of the independent flight phases. It has six degrees of freedom, out of which four are independent (heave, pitch, roll, yaw) and two are coupled (surge and sway). Hence, it is not possible to achieve surge and sway motion independently without any roll and pitch input. The position and attitude of the entire hybrid VTOL tilt-rotor is controlled by continuously varying the collective angular velocity and the deflection of the thrusters. As the entire flight envelope is divided into three definitive phases, i.e., the VTOL hover phase, the transition phase, and the fixed-wing cruise phase, the working principle is summarized in each of the independent phases as mentioned below.

**Figure 4:**
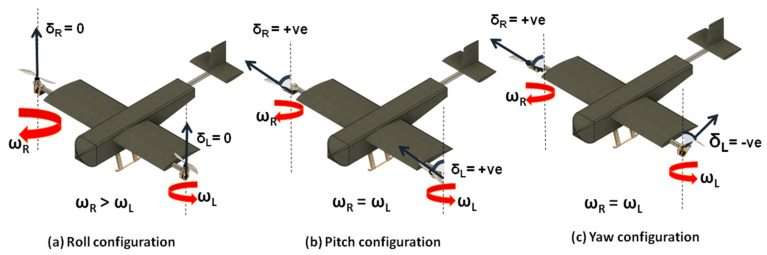
Isometric view of tilt-rotor during yaw, roll, and pitch in the VTOL flight phase. Here, (**a**) depicts the roll configuration due to differential thrust, (**b**) depicts the pitch configuration due to tilting of the motor-pod and (**c**) illustrates yaw due to tilting of the motor-pod in opposite directions.

(1) VTOL or Hover Phase: During takeoff, landing, and hovering motion, the hybrid platform is in the VTOL phase. In this phase, the thrusters (combination of actuator and propeller) are in the vertical orientation. Heave motion with an axis pointing onto the plane, as shown in Figure 1, is achieved by increasing or decreasing the angular velocity of the two rotors proportionately. Roll motion (coupled with sway motion) is achieved by increasing the angular velocity of one thruster with respect to the other thruster. Hence roll right motion is obtained by increasing the angular velocity of the left thruster with respect to the right thruster. Pitch and yaw motion are a more challenging, as they are not only controlled by varying the angular velocity of the thrusters but also by the deflection of the individual motor pods. Pitch-up motion is achieved by tilting the thruster towards the nose, which creates a moment and a horizontal component of thrust force, leading to surge motion. Similarly, yaw motion is generated by tilting the actuator in opposite directions, which creates a couple of CG due to the horizontal component of the thrust being directed in opposite directions for both the thrusters. A representative figure demonstrating the working of the hybrid VTOL tilt-rotor in each independent flight phase is shown in Figure 4.

(2) Transition Phase: After attaining a particular altitude, the vehicle undergoes a transition where both of the rotors change their orientation from a vertical alignment to a horizontal alignment. As in the VTOL phase, the vehicle’s entire weight is supported by the collective thrust of the actuators, while in the fixed-wing phase, the lift from the wing accounts for the weight. Hence during the transition, a slight dip in altitude is expected, and the key idea to achieve a seamless transfer from one flight mode to another is to make the transition slow, such that by the time the thrusters have horizontal alignment, the hybrid UAV would have attained sufficient horizontal velocity to produce the required lift from the wing to keep the platform airborne. The slight dip in altitude is introduced because of the time interval required for change in thruster direction, which eventually leads to a change in the thrust direction from the vertical heave axis to the horizontal surge axis. Thrust vectoring reduces the actuator force acting along the heave axis, leading to a dip in altitude, which is, again, an actuator limitation, as discussed later in the simulation section.

(3) Fixed-Wing or Cruise Phase: In this flight phase, both thrusters are in horizontal alignment, and the working principle is similar to that of a conventional aircraft. Heave motion or variation in altitude is achieved by varying the angular velocity of the thrusters, which, in turn, affects the lift generated from the wing. Roll motion is controlled by combined input from the ailerons and varying the angular velocity of the rotors, while pitch and yaw motion is controlled by deflecting the elevator and rudder surfaces, respectively. The overall working of the hybrid VTOL tilt-rotor in the three different flight phases is summarized in Table 2. In the summarized table, the right motor is labeled as M1, and the left motor is labeled as M2, as shown in Figure 2, whereas their angular velocity is denoted by *ω_R_* (clockwise) and *ω_L_* (counterclockwise). A positive tilt of the motor pod denotes the tilting of the actuator towards the nose of the aircraft.

## 4. Mathematical Modelling

In this section, we develop the mathematical model of the hybrid VTOL tilt-rotor based on the forces and moments acting on it. Understanding the dynamics of such a hybrid vehicle not only helps us to formulate a reliable and accurate mathematical model for the vehicle, capturing the complete information of the vehicle’s pose (position and orientation), but also helps in the development of robust flight control algorithms.

Formulating a unified dynamic model for the hybrid vehicle provides the distinct advantage of modeling the entire flight envelope as a continuous flight regime, ruling out the complexity of flight mode switching between two discrete flight profiles and their dynamic models.

### 4.1. Kinematic Modeling of the Bi-Rotor

For the kinematic modeling of the hybrid VTOL aerial vehicle, two coordinate frames of reference are defined [20], as illustrated in Figure 2:

(1) Body fixed frame of reference (X_B_, Y_B_, and Z_B_).
(2) Earth fixed inertial frame of reference (X_E_, Y_E_, and Z_E_).

The position and attitude of the center of mass of the vehicle with respect to the inertial frame of reference is given by

(1) $$\eta_{1}={\left[x\;y\;z\right]}^{T},\;\eta_{2}={\left[\Phi \;\theta \;\Psi \right]}^{T}$$

where Φ, θ, and Ψ are the roll, pitch, and yaw angles, respectively, denoting the orientation of the center of mass using Euler angle representation. The complete pose of the center of mass of the hybrid vehicle is represented by combining both the position and attitude vectors.

(2) $$\eta_{E}={\left[{\eta_{1}}^{T}\;{\eta_{2}}^{T}\right]}^{T}$$

Similarly, any change in the pose of the overall system with the CG at **η_E_** would lead to velocity in the inertial frame of reference, which is represented by

(3) $${\overset{\cdot }{\eta }}_{E}={\left[{{\overset{\cdot }{\eta }}_{1}}^{T}\;{{\overset{\cdot }{\eta }}_{2}}^{T}\right]}^{T}$$

where ${\overset{\cdot }{\eta }}_{1}$ represents the linear velocity, and ${\overset{\cdot }{\eta }}_{2}$ represents the angular velocity of the vehicle with respect to the inertial frame of reference. However, the velocity vector of any vehicle is measured by sensors mounted in the body reference frame, which can be expressed as

(4) $$V_{B}={\left[{V_{1}}^{T}\;{V_{2}}^{T}\right]}^{T}$$

where $V_{1}={\left[V_{x}\;V_{y}\;V_{z}\right]}^{T}$ and $V_{2}={\left[p\;q\;r\right]}^{T}$ denote the linear and angular velocity measured in the body-fixed frame of reference.

The kinematic relationship between the velocities in the inertial frame and body-fixed frame of reference is given by the following equation:

(5) $${\overset{\cdot }{\eta }}_{E}=J(\eta_{2})\cdot V_{B}$$

which can be rewritten in the matrix form as shown below:

(6) $$\left[\begin{array}{c} {\overset{\cdot }{\eta }}_{1}\\ {\overset{\cdot }{\eta }}_{2} \end{array}\right]=\left[\;\begin{array}{cc} J_{1}\left(\eta_{2}\right) & 0_{3x3}\\ 0_{3x3} & J_{2}\left(\eta_{2}\right)\; \end{array}\right].\;\left[\begin{array}{c} V_{1}\\ V_{2} \end{array}\right]$$

Here J(η_2_) is the Jacobian matrix mapping linear and angular velocity in the body-fixed frame to the inertial frame of reference and is decomposed into J_1_(η_2_) to transform the linear velocity, J_2_(η_2_) to transform the angular velocity, and $0_{3x3}$, which is a null matrix. The individual decomposed Jacobian matrices J_1_(η_2_) and J_2_(η_2_) can be expanded as follows:

(7) $$J_{1}\left(\eta_{2}\right)=\left[\begin{array}{ccc} c\Phi c\theta \; & -s\Psi c\Phi +c\Psi s\theta s\Phi \;\; & s\Psi s\Phi +c\Psi c\Phi s\theta \\ s\Phi c\theta & c\Psi c\Phi +s\Phi s\theta s\Psi & -c\Psi s\Phi +s\theta s\Phi c\Phi \\ -s\theta & c\theta s\Phi & c\theta c\Phi \end{array}\right]\;$$

(8) $$J_{2}\left(\eta_{2}\right)=1/c\theta \left[\begin{array}{ccc} 1\; & s\Phi s\theta \;\; & c\Phi s\theta \\ 0 & c\Phi c\theta & -s\Phi c\theta \\ 0 & s\Phi & c\Phi \end{array}\right]\;$$

where the abbreviations sβ and cβ are used instead of ***sin***(β ) and ***cos***(β ), respectively.

### 4.2. Dynamic Modeling of the Bi-Rotor

The dynamics of the hybrid VTOL bi-rotor is represented by a unified dynamic model, which includes horizontal level flight utilizing the aerodynamic lift and drag forces produced by the wing, vertical flight using the thrust generated by the two actuators, and the transition phase, which incorporates a mix of horizontal and vertical flight dynamics.

The non-linear dynamic equations obtained for the hybrid aerial vehicle are derived considering the following assumptions:

(a) The entire airframe is a rigid body, which implies that the distance between any two points on the aircraft does not change, which also applies to the propellers.
(b) The rotation of the earth is negligible in comparison to the acceleration of the aerial vehicle, making the earth a fixed frame of reference for the inertial reference frame.
(c) In the body-fixed frame, X_B_Z_B_ is the plane of symmetry, making the off-diagonal terms in the inertial matrix equal to zero.
(d) The tilt of the motor pods (*δ**r***, *δ**l***) does not affect the mass distribution of the vehicle. Hence, the mass and inertial terms remain unchanged, as expressed in Table 1.

Here, the 6 DOF dynamic equations for the aerial vehicle in the body-fixed frame of reference is expressed as per the Newton–Euler formulation as shown [21]:

(9) $$F_{ext}=m{\dot{V}}_{B}+\omega_{B}\times \left(mV_{B}\right)$$

(10) $$M_{ext}=I{\dot{\omega }}_{B}+\omega_{B}\times \left(I\omega_{B}\right)$$

where $F_{ext}$ and $M_{ext}$ are the external forces and moments acting on the CG, *m* is the overall mass of the airframe, and *I* is the inertial matrix expressed in the body fixed frame of reference. The total external forces and moments acting on the body can be decomposed into a sum of forces and moments generated by individual components, as shown in the following equation:

(11) $$F_{ext}=F_{thrusters}+F_{gravity}+L_{wing}+D+F_{dist}$$

(12) $$M_{ext}=M_{thrusters}+M_{gyro}+M_{aero}+M_{consurf}$$

The total external force acting on the body is expressed as the sum of the following forces: $F_{gravity}$ is acting on the center of gravity, $F_{thrusters}$ is generated by the rotors acting on the point of actuation, which happens to be at the end of nacelles in our case, $L_{wing}$ is the lift force generated by the wing, and D is the total drag force generated by the aircraft. Due to the external gust disturbances, the force contribution have also been modeled as $F_{dist}$. Similarly, the external moment is expressed as the sum of the torques $M_{thrusters}$ created by the rotors, the $M_{aero}$ created by the moment due to lift and drag forces generated by the wing about the CG, the $M_{consurf}\;,$ which is the moment generated due to the deflection of control surfaces, i.e., the ailerons, rudders, and elevators, which are effective only in the fixed-wing flight phase, and $M_{gyro}$, which is created by the gyroscopic effect of the propellers. An important point to note here is that the interference between the motor pod and the propellers are neglected because the point of actuation is at the end of the wing boom. Additionally, as the major forces considered in the fixed-wing flight phase are the lift and drag forces, it is safe to consider that the aerodynamic interference has a negligible effect on these terms. However, these aerodynamic interactions can be studied by wake vortex or velocity distribution plots in CFD simulation [22] or experimentally by a load cell inside the wind tunnel setup [23].

Forces due to gravity and thrusters are intuitive and easy to grasp. While gravity remains along the *Z*-axis, being multiplied with the coordinate transformation matrix to transform it from the inertial frame of reference to a body-fixed frame of reference, other forces need an analytical derivation based on the corresponding frames and the associated aerodynamic coefficients.

(13) $$F_{gravity}=\left[\begin{array}{ccc} 1 & 0 & -sin\theta \\ 0 & cos\Phi & sin\Phi \cdot \cos\theta \\ 0 & -sin\Phi & cos\Phi \cdot cos\theta \end{array}\right]\left[\begin{array}{c} 0\\ 0\\ mg \end{array}\right]$$

(14) $$\;\;\;\;\;\;\;\;\;\;\;\;\;F_{gravity=}\left[\begin{array}{c} -mgsin\theta \\ mgsin\Phi \cdot cos\theta \\ mgcos\Phi \cdot cos\theta \end{array}\right]$$

In a similar fashion, forces due to thrusters are multiplied with the rotational transformation matrix, which is defined by the tilt angle of the motor pod at the ***Y***-axis, as shown in Figure 2. The tilt angle is denoted by $\delta_{r}$ for the right motor pod and by $\delta_{l}\;$for the left motor pod, respectively.

(15) $$\;F_{thrusters}=\left[\begin{array}{ccc} cos\delta_{r} & 0 & sin\delta_{r}\\ 0 & 1 & 0\\ -sin\delta_{r} & 0 & cos\delta_{r} \end{array}\right]\left[\begin{array}{c} 0\\ 0\\ -Fr \end{array}\right]+\left[\begin{array}{ccc} cos\delta_{l}\; & 0 & sin\delta_{l}\;\\ 0 & 1 & 0\\ -sin\delta_{l}\; & 0 & cos\delta_{l}\; \end{array}\right]\left[\begin{array}{c} 0\\ 0\\ -Fl \end{array}\right]\;\;$$

(16) $$F_{thrusters}=\left[\begin{array}{c} -Fr\left(sin\delta_{r}\right)-Fl\left(sin\delta_{l}\right)\\ 0\\ -Fr\left(cos\delta_{r}\right)-Fl\left(cos\delta_{l}\right) \end{array}\right]$$

In the VTOL flight phase, the effect of aerodynamic forces acting on the hybrid aerial vehicle is negligible, but consecutively with the tilt of motor pods, the wings generate aerodynamic lift and drag force, which can be expressed by the equation [20]:

(17) $$\left[\begin{array}{c} D_{wing}\\ 0\\ L_{wing} \end{array}\right]=R\left(\alpha_{i}\right)\;\left[\begin{array}{c} -qSC_{D}\left(\alpha \right)\\ 0\\ -qSC_{L}\left(\alpha \right) \end{array}\right]$$

Here, $R\left(\alpha_{i}\right)$ is the rotational transformation matrix used to transform the lift and drag forces back to the body-fixed frame of reference, S is the wing planform area, and $C_{D}\left(\alpha \right)$ and $C_{L}\left(\alpha \right)$ are the lift and drag coefficient of the DAE 51 airfoil. Here, the dynamic pressure (*q*), as illustrated in Equation (17), can be expressed in terms of the density of air (ρ) and the airstream velocity$\;\left(V_{\infty }\right)\;$as: $q=\frac{1}{2}\rho V_{\infty }^{2}$.

**Figure 5:**
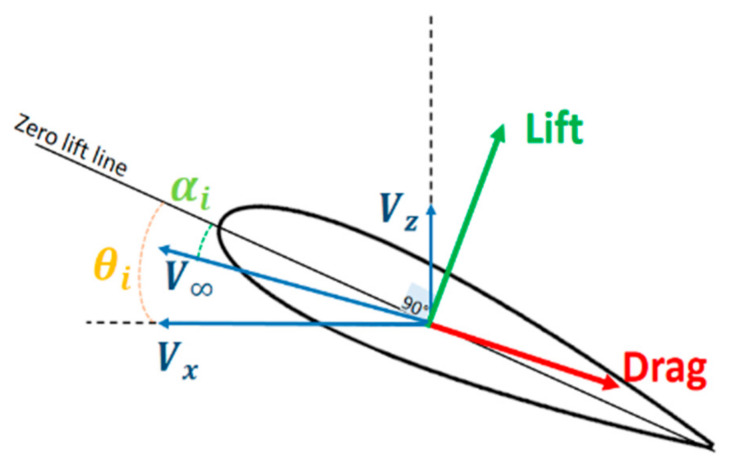
Representative figure illustrating the effective angle of attack.

The resultant airstream velocity ($V_{\infty }$) can be expressed in terms of ascending ($V_{z}$) and cruising velocity ($V_{x}$) as: $V_{\infty }=\sqrt{{\left(V_{x}\right)}^{2}+{\left(V_{z}\right)}^{2}}$, and the effective angle of attack can be expressed in terms of velocity and the tilt angle, $\alpha_{i}=\theta_{i}-\left(atan2\left(V_{z}/V_{x}\right)\right)$, as illustrated in Figure 5.

Lift and drag forces are dependent on the coefficient of lift and drag, respectively, and with varying angles of attack in the hybrid VTOL tilt-rotor, there should be an intuitive change in these coefficients, too. In conventional fixed-wing aircrafts, both $\;C_{L}$, $C_{D}$ are plotted for the fixed angle of flight during cruise conditions. In a rotary wing, blade element theory is utilized to compute the variation in the lift and drag. However, in rotary wings, the variation of the angle of attack is less, as the rotary-wing assumes pre-stall conditions during flight. Hence, using the conventional mathematical models to find the variation of $C_{L}$ and $C_{D}$ will not work because of the significant change in angle of attack. Additionally, lift and drag are not only the functions of incident angle of attack and longitudinal velocity but are also dependent on the vertical ascending and descending velocity.

**Figure 6:**
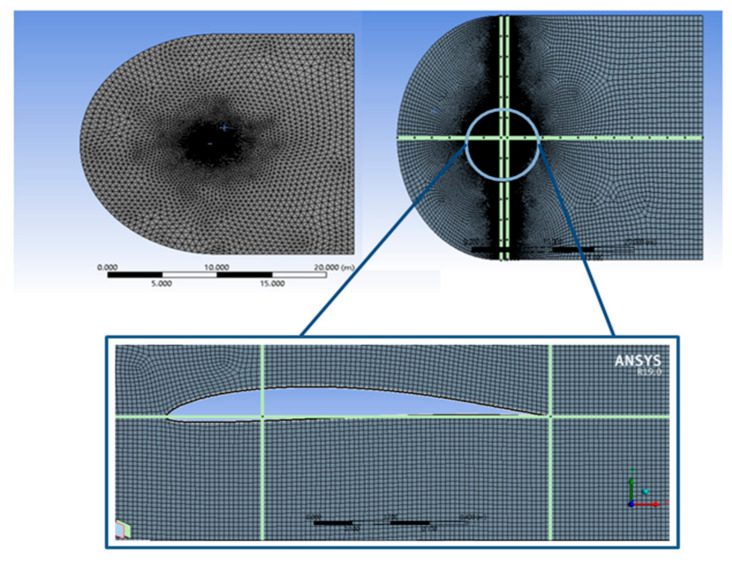
Mesh generated in the fluid medium for DAE 51 aerofoil.

To analyse this, the ANSYS simulation environment was used to simulate the flight of the aerofoil for variations in the angle of attack. Initially, the fluid medium was discretized with C-type mesh [24], as shown in Figure 6.

**Figure 7:**
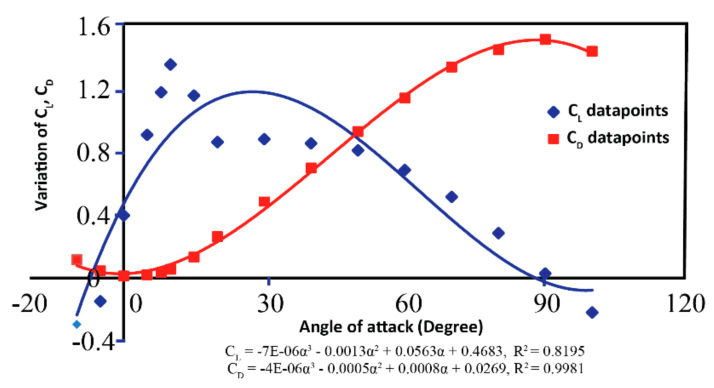
Variation of${\;C}_{L\;}$ and${\;C}_{D}$ with change in angle of attack.

After meshing, the boundary conditions are defined where the incoming edge of the mesh is defined as the velocity inlet, and the rear end is the pressure outlet. Corresponding solutions are determined for every design point by varying the effective angle of attack in each iteration from −10° to 110° where the mesh motion is set to 0.5 rad/s. The solution plot signifying the variation of $C_{L\;}$ and $C_{D}\;$with changes in the attack angle is shown in Figure 7.

**Figure 8:**
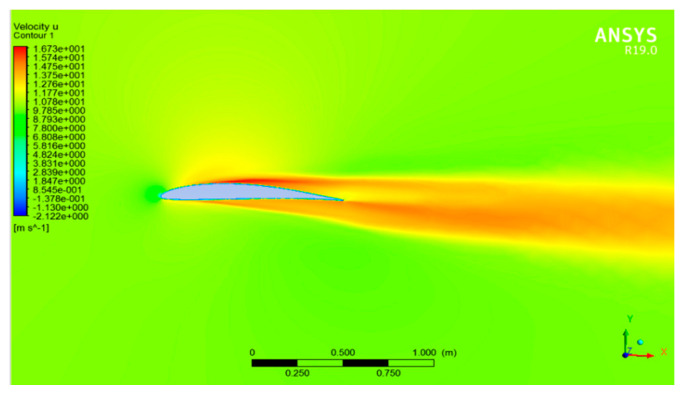
Contour plot of velocity vector for cruise conditions (15 m/s) at zero AOA.

To validate the solution, one of the velocity contour plots for DAE 51 airfoil in cruise conditions is shown in Figure 8, which depicts a positive pressure potential on the bottom of the aerofoil.

**Figure 9:**
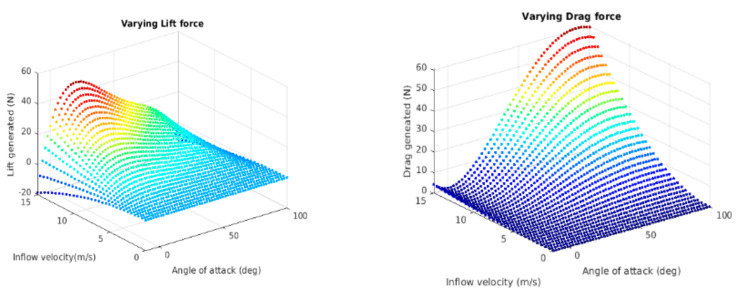
Variation of lift and drag forces with change in the AOA and airflow velocity.

The variation of the lift and drag forces with the change in angle of attack (0–100°) and airstream velocity (0–15 m/s) is captured in Figure 9. As seen in the figure, a positive lift force is generated in the fixed-wing flight phase, and a positive drag force is generated at the VTOL phase due to changes in the angle of attack from 0° to 90°.

Finally, the wind and gust disturbances are modeled as three-dimensional random sources (3 × 1) in the Simulink environment, which provides random forces for each interval in simulation time, bounded within a magnitude of 0.1 N.

The moment generated by the thrusters is represented by $M_{thrusters}$, which can be mathematically modelled as:

(18) $$M_{thrusters}={\left(\vec{r}\;x\;\vec{F}\right)}_{rotor1}+{\left(\vec{r}\;x\;\vec{F}\right)}_{rotor2}$$

Here, $\vec{r}$ is the position vector of the point of the actuation from the origin, and $\vec{F}$ is the force generated by the thrusters. Here, the position vector points to the end of the wing boom, which simplifies the equation into:

(19) $$\;M_{thrusters}=\left[\begin{array}{ccc} 0 & -z & y\\ z & 0 & -x\\ -y & x & 0 \end{array}\right]\left[\begin{array}{c} -Frsin\delta r\\ 0\\ -Frcos\delta r \end{array}\right]+\left[\begin{array}{ccc} 0\; & -z & y\;\\ z & 0 & -x\\ -y\; & x & 0\; \end{array}\right]\left[\begin{array}{c} -Flsin\delta l\\ 0\\ -Flcos\delta l \end{array}\right]$$

where *x* = 0.10 m, *y* = *b*/2, and *z* = 0 at the point of the actuation, which is displaced at the ends of the wing boom and is shifted from the CG by the semi wingspan length (*b*/2).

The aerodynamic moments represented by $M_{aero\;}$ occur due to an imbalance of the lift and drag forces from the CG. However, due to the assumption that both the CG and CP coincide with a little deviation along the x coordinates, the aerodynamic moments can be considered to be negligible.

Gyroscopic torque arises when there is a change in the rotation plane of a rotating object. In our case, during the transition, there is a tilt in the motor pods, which leads to the gyroscopic couple acting on the airframe [4], which can be modelled as:

(20) $$M_{gyro}=\overset{2}{\underset{i=1}{\sum }}I_{rotor}\left(n_{i\;}\omega_{b}\times \left[\begin{array}{c} c\theta_{i}\\ 0\\ -s\theta_{i} \end{array}\right]\;\omega_{i}\right),\;n_{i\;}=1,-1$$

where $I_{rotor}$ is the moment of inertia of the motor pod at the rotor axis, $\omega_{b}$ is the rate of change of the motor pod angle, and $\omega_{i}$ is the angular velocity of the rotors.

For a small-scaled UAV moment generated by the control surfaces, it can be expressed in terms of wingspan (*b*), aerodynamic chord ($\bar{c}$), and reference area (*S*) in a similar fashion as the lift and drag forces are derived earlier:

(21) $$M_{consurf}=\left[\begin{array}{c} bC_{l}qS\\ \bar{c}C_{m}qS\\ bC_{n}qS \end{array}\right]\;$$

where, q is the dynamic pressure and$\;C_{l}$, $C_{m}$, and $C_{n}$ are the roll, pitch, and yaw aerodynamic coefficients. Here, the roll, pitch, and yaw coefficients can also be expanded as [25]:

(22) $$C_{l}=C_{l0}+C_{l\beta }\beta +C_{l\alpha }\alpha +C_{l\delta_{a}}\delta_{a}+C_{lp}\frac{b}{2V_{\infty }}p+C_{l\delta_{r}}\delta_{r}$$

(23) $$C_{m}=C_{m0}+C_{m\alpha }\alpha +C_{m\delta_{e}}\delta_{e}+C_{mq}\frac{c}{2V_{\infty }}q$$

(24) $$C_{n}=C_{n0}+C_{n\beta }\beta +C_{n\delta_{r}}\delta_{r}+C_{nr}\frac{b}{2V_{\infty }}r+C_{n\delta_{a}}\delta_{a}\;$$

Here, as shown in Equations (22)–(24), the moment coefficients depend on aileron defection ($\delta_{a}$), elevator deflection ($\delta_{e}$), rudder deflection ($\delta_{r}$), the angular velocity of the body (p, *q*, *r*), the angle of attack (*α*), the sideslip angle (β), and the airspeed velocity ($V_{\infty }$). Not only do the control surfaces have an effect on the moment coefficients, but they also have an overall effect on the lift and drag of the airframe. Considering elevators as an example, the down pitching of an elevator increases the airfoil’s asymmetry, leading to an increase in the lift coefficient. There is also an effect on the angular velocity of the vehicle on the moment coefficients denoted by $C_{lp},\;C_{mq},\;\mathrm{and}\;C_{nr}.\;$From the above equations, it is clear that the bi-rotor’s roll and yaw behavior are coupled as a deflection in the aileron effects on the yawing coefficient, with the opposite only being valid in the fixed-wing flight phase.

Generally, the aerodynamic parameters for control surface deflections are derived from wind tunnel tests or CFD simulations. Considering the difficulties in the CFD and wind tunnel tests, the estimation of the control derivatives for the VTOL tilt-rotor is achieved by utilizing XFLR, which was introduced by MIT in 2019 [18].

**Figure 10:**
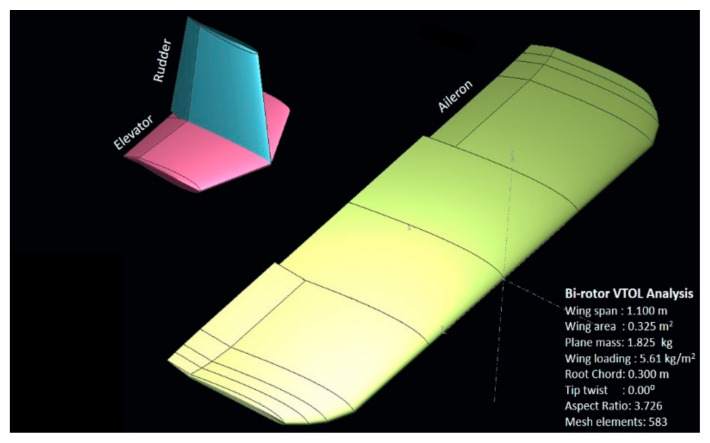
Hybrid VTOL tilt-rotor modeled in XFLR.

**Figure 11:**
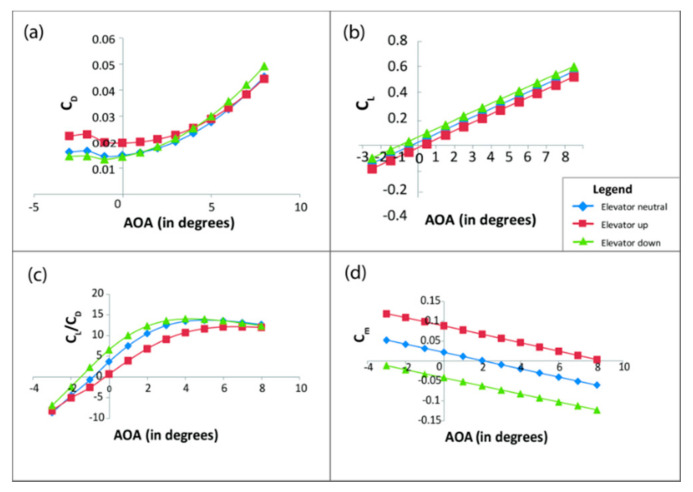
Change in aerodynamic parameters due to elevator deflection. Here, subfigure (**a**) shows the variation of C_D_ (Coefficient of drag), (**b**) shows the variation of C_L_ (Coefficient of lift), (**c**) shows the variation of C_L_/C_D_, and (**d**) shows the variation of C_m_ (Coefficient of pitching moment) with varying angle of attack and fixed elevator deflection of 10°.

**Figure 12:**
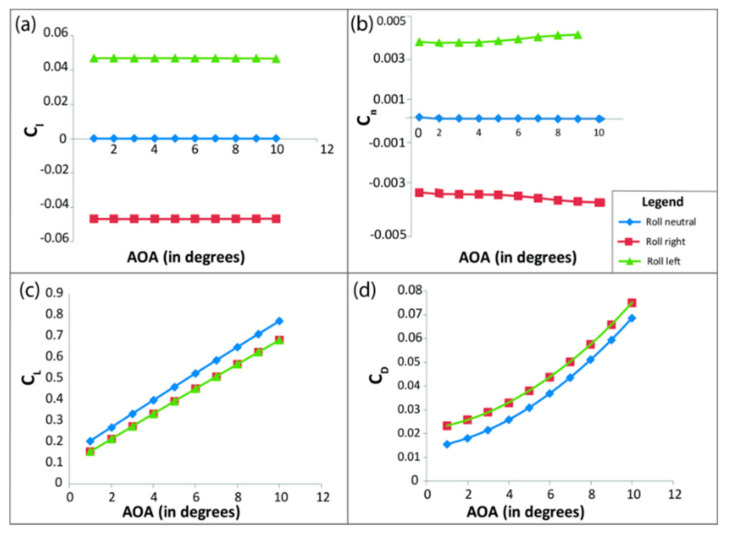
Change in aerodynamic parameters due to aileron deflection. Here, subfigure (**a**) shows the variation of C_l_ (coefficient of pitching moment), (**b**) shows the variation of C_n_ (coefficient of the yawing moment), (**c**) shows the variation of C_L_ (coefficient of lift), and (**d**) shows the variation of C_D_ (coefficient of drag) with varying angles of attack and a fixed aileron deflection of 10°.

**Figure 13:**
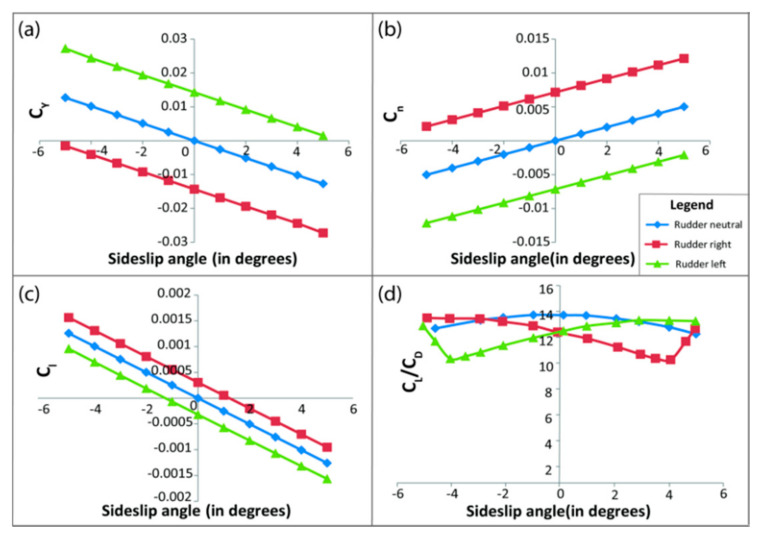
Change in aerodynamic parameters due to rudder deflection. Here, subfigure (**a**) shows the variation of C_Y_ (coefficient of side force), (**b**) shows the variation of C_n_ (coefficient of yawing moment), (**c**) shows the variation of C_l_ (coefficient of rolling moment), and (**d**) shows the variation of C_L_/C_D_ with varying sideslip angles and a fixed rudder deflection of 10°.

After modeling the entire VTOL tilt-rotor in XFLR (Figure 10) with a similar airfoil profile and mass distribution, the tilt-rotor model, as shown, is simulated for different control surface deflections (varying from −10 degrees to 10 degrees), varying both the AOA and sideslip angle (varying from −10 to 10 degrees) to estimate the aerodynamic coefficients, and the results are as shown in Figure 11, Figure 12 and Figure 13.

Considering Figure 11, which highlights the effect of aerodynamic coefficients due to elevator deflection, here, we observe that the elevator down configuration leads to an increase in asymmetry in the airfoil. This asymmetry or camber in the airfoil further leads to an increase in the lift coefficient (C_L_), and drag being induced by lift also increases the drag coefficient (C_D_). Finally, as shown in the subfigure (d), the elevator down configuration induces an anticlockwise moment on the hybrid aircraft, which is considered negative, while a positive counterclockwise moment is created by the elevator up configuration.

Similarly, Figure 12 and Figure 13 show the effect of aileron and rudder on various aerodynamic parameters. Here, the effects of rudder deflection are studied based on varying sideslip deflection, unlike the elevator and ailerons. These varying aerodynamic coefficients are further imported to the Simulink interpolation block for dynamic modelling

The main difference that can be noticed from such an analysis is that in the neutral configuration, the change in the aerodynamic coefficients is negligible. Hence, unlike the conventional fixed-wing vehicles, the tilt-rotor utilizes the differential actuation and tilt of the motor pods to accommodate the loss in degree of freedom due to the two actuators in the neutral VTOL configuration, while in the fixed-wing configuration, the control surface is effective for a change in the orientation of the hybrid aerial vehicle, as shown in the XFLR simulations. Conventionally the aerodynamic parameters are derived experimentally from wind tunnel experiments [26], analytically by DATCOM [27], or from computational simulations which is implemented in this paper. 

## 5. Simulation and Analysis

**Figure 14:**
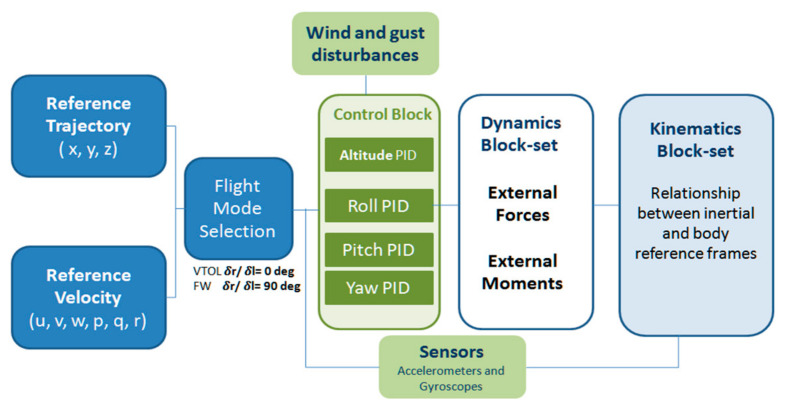
System model of hybrid VTOL tilt-rotor.

After modeling the non-linear dynamics in the MATLAB Simulink environment, the next step was to implement the control architecture on the hybrid VTOL tilt rotor. The control architecture’s ability to achieve steady flight while maintaining the desired altitude and attitude would help us verify the robustness of the flight control algorithm on the hybrid vehicle. The control architecture, as shown in Figure 14, utilizes four standard PID controller blocks to stabilize altitude, roll, pitch, and yaw. The four virtual outputs of the control blocks (u1, u2, u3, u4), which can be compared to the four channels input in conventional miniaturized UAV, i.e., throttle, aileron, elevator, and rudder is further fed into the flight mixer block. The flight mixing block then redistributes the four virtual control inputs into seven actuator inputs, which are the angular velocity of the rotor (***ωr***, ***ωl***), motor tilt (***δ***_right_, ***δ***_left_), and control surface deflection (***δe***, ***δa***, ***δr***). For example, the output u1, which is the output of the altitude controller block, translates to a change in the angular velocity of both the rotors in VTOL mode but, on the other hand, also leads to elevator deflection in fixed-wing flight mode.

**Figure 15:**
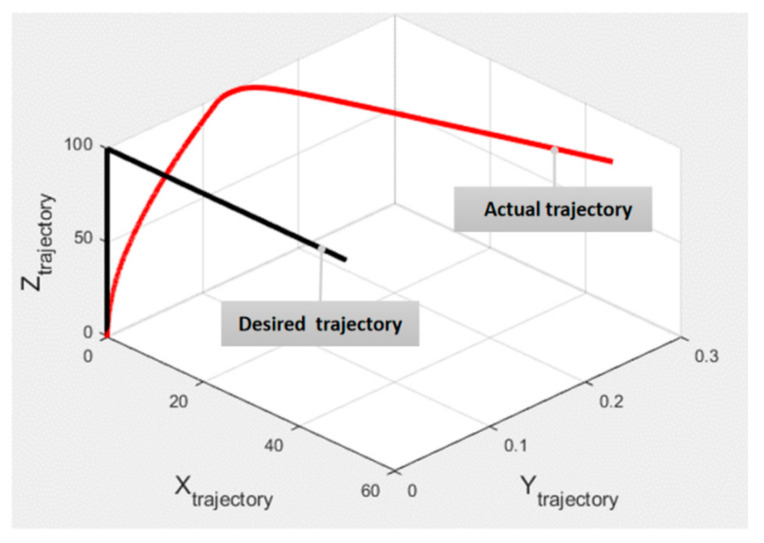
Trajectory tracking of hybrid VTOL vehicle.

The flight mixing block is responsible for mapping the virtual control inputs into actuator signals, which are fed into the dynamic model block visualized by the HL20 VR Simulink module [28]. The performance of the dynamic model with the implemented control system architecture is verified by simulating trajectory tracking for a step response where the model is able to track the desired trajectory with a maximum permissible error of 0.25 m, as shown in Figure 15.

**Figure 16:**
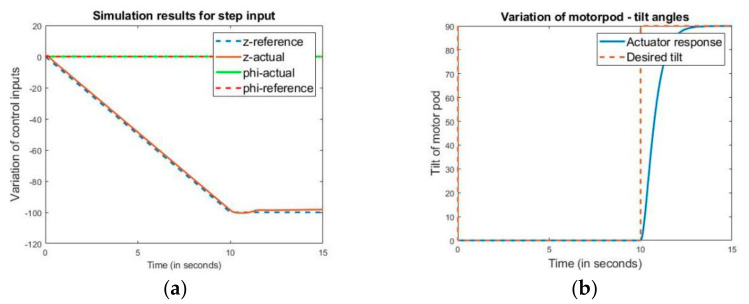
Variation of control parameters in the flight profile. Here, (**a**) shows the variation of the height and pitch angle along with the flight profile, and (**b**) shows the tilt of both the motor pods during the entire flight profile.

Similarly, Figure 16 depicts the attitude of the hybrid vehicle and controller input for the motor pods while tracking the given reference trajectory.

As shown in Figure 16b, the motor pod undergoes a tilt from 0° to 90°, performing a transition from the VTOL flight phase to the fixed-wing phase, which can also be verified by the dip in altitude. The tracking of the reference trajectory within the permissible error of 0.25 m while maintaining the specific altitude and attitude verifies the controller’s robustness and feasibility.

## 6. Experimental Trials

**Figure 17:**
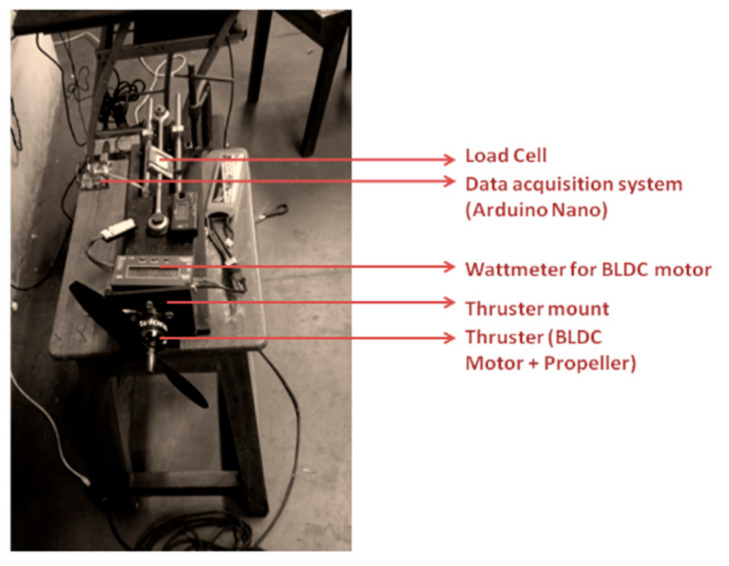
Experimental load cell setup for thrust calculation.

An experimental investigation of the individual subsystem was conducted before assembling the prototype of the hybrid vehicle. Preliminary tests were conducted on the propulsion subsystem, which includes the propeller and the actuator setup. These tests were performed to determine the stable operating RPM and to verify whether the overall thrust to weight ratio for the vehicle is greater than one. The experimental setup, as shown in Figure 17, consists of the thruster mounted on the load cell incorporated with the HX711 load cell amplifier [29]. A data acquisition system (DAS) built on the Arduino Nano platform was used for data collection. Simultaneously, the current and voltage readings from the wattmeter were also recorded to notice the overall power consumption.

The setup was used to analyse the 2-bladed APC 9″ × 4.5″ propellers and the 3-bladed CFRP 11″ × 3″ propellers mounted on the 1100 KV DC brushless motor and the 14.6V Li-Po battery for the power supply. At stable operating conditions of 5000 rpm, 11″ × 3″, propellers generated 1.28 kgf thrust with a power consumption of 799.68 W. On the other hand, the 9″ × 4.5″ propellers generated 1.44 kgf thrust while consuming 585.4 W.

Hence, 9″ × 4.5″ CFRP propellers were selected for the prototype, which produced an approximate thrust to weight ratio of 1.5 and a specific thrust ratio (gram/weight), which is better in comparison to a conventional UAV [30]. Power consumption was also analysed and was compared with that of the conventional quad-rotor and tri-rotor platforms in the existing literature [31], where the hovering efficiency is analytically derived from momentum theory. It was concluded that out of all the 12 available conventional aerial platforms, bi-rotors are compact yet efficient because of their low disk loading and greater hovering efficiency.

The design implemented here also incorporates the fixed-wing aspect to account for the lift of the wing, which will lead to an increase in endurance without compromising the power requirements. One of the intuitive reasons for bi-rotors being an efficient approach in aerial flight is the smaller number of actuators, resulting in less inherent power loss associated with each and every component, leading to the greater efficiency of the overall system. Apart from the analytical studies in [31,32], performed experimental static thrust tests also verified the ability of the propulsion subsystem to utilize less power in comparison to the conventional quad-rotor platform.

**Figure 18:**
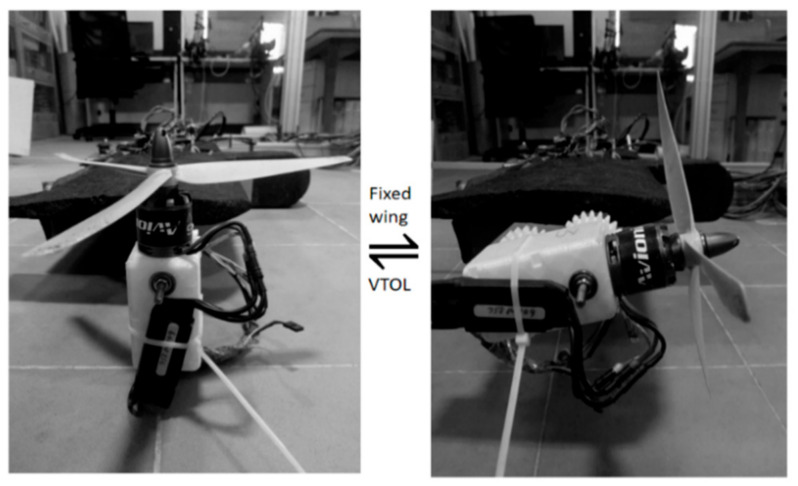
Fabricated prototype of the motor pod (Version1).

**Figure 19:**
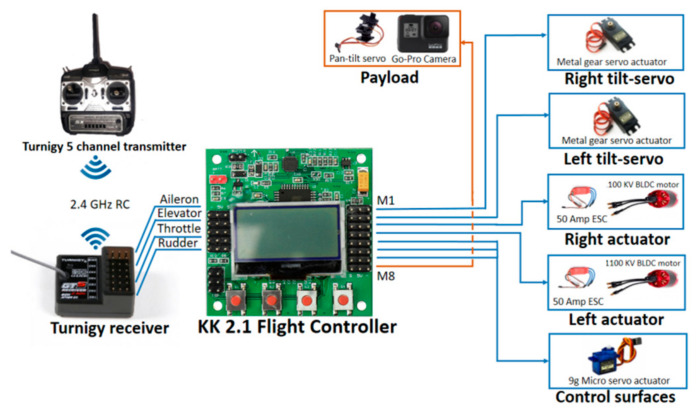
Schematic hardware implementation diagram.

The initial test platform undergoing a flight transition from VTOL to a fixed-wing phase is shown in Figure 18. After the proper selection of the individual hardware components such as the flight controller [33], propellers, motors, and ESC, the schematic diagram was determined, as shown in Figure 19.

**Figure 20:**
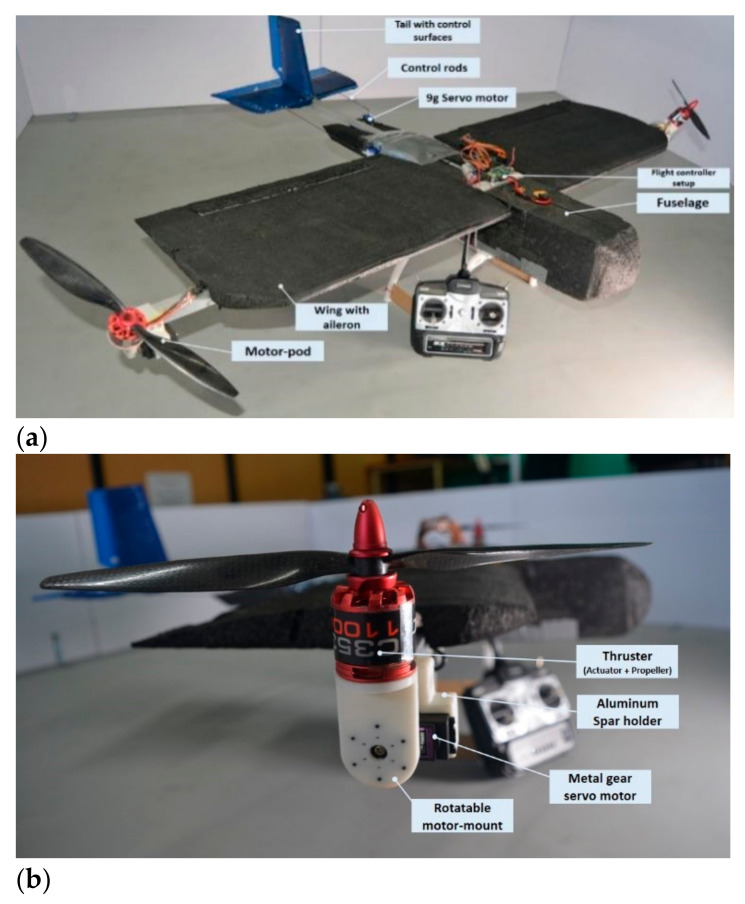
(**a**) Fabricated prototype of Hybrid VTOL tilt-rotor laying down the individual components and (**b**) the fabricated prototype of motor pod.

The hardware components were laid out as mentioned in the schematic and some minor modifications were made to the initial test platform, and the final prototype with the individual components is laid out in Figure 20. The prototype fabrication was made using polystyrene foam sheets, square aluminum rods, and custom 3D printed ABS mounts. 

Preliminary hover trials were conducted on the experimental prototype for an average flight time of 1 min, which verified stable hover ability at an altitude of half a meter above the ground, but the flight transitions induced pitch instability.

## 7. Conclusions and Future Work

This paper has presented a novel design of a hybrid fixed-wing bi-copter with thrust vectoring capabilities to overcome the range and endurance limitations of rotorcraft configuration. The novel design of the tilting motor pod allowed a smooth transition between the fixed-wing and VTOL phases, robust control over yaw, and pitch motions without any need for additional actuators during the VTOL hover phase. The computational simulations helped us to approximate the mathematical model that was further verified using dynamic modeling in MATLAB. The control algorithm implemented on the dynamic model verified the stability of the hybrid UAV in the fixed-wing, transition, and VTOL phases. In the experimental trials, we have verified the stability of the hybrid vehicle in hover conditions. In future works, we will explore the possibility of optimizing the angle of attack to maximize the cruise distance, implementing autonomous flight switching algorithms using better flight controllers to transition from hover to fixed-wing flight, and performing extensive trials to achieve stable operating conditions while transitioning between flight modes.

## Figures and Tables

**Table 1:** Mechanical design parameters specification.

Parameter	Specified Value
Configuration	VTOL Tilt rotor
Wing Span length	110 cm
Wing chord length	30 cm
Aero foil	DAE 51
Overall weight/length/breadth	1.8 kg/110 cm/100 cm
Propeller diameter and pitch	2 bladed 12″ × 5″
Centre of gravity	39 cm from nose of the aircraft
Inertia (I_xx_, I_yy_, I_zz_)	0.1365 kg·m^2^, 0.04401 kg·m^2^, 0.1802 kg·m^2^
Moment of inertia of the rotor-pod (I_rotor_)	0.050 kg·m^2^

**Table 2:** Control inputs for various flight phases in hybrid VTOL tilt-rotor.

Flight Phase	Desired Motion	M1 Tilt	M2 Tilt	Relation between *ω_R_* and *ω_L_*	Control Surface Input
VTOL	Heave	0°	0°	Increase or decrease proportionally *ω_R_* = *ω_L_*	Nil
	Roll	0°	0°	Differential increase in angular velocity *ω_R_* > *ω_L_* or *ω_L_* > *ω_R_*	Nil
	Pitch up	+ve	+ve	Same angular velocity in both thrusters *ω_R_* = *ω_L_*	Nil
	Yaw	−ve	+ve	Same angular velocity in both thrusters *ω_R_* = *ω_L_*	Nil
Transition	Changing orientation of rotors	90°	90°	Same angular velocity in both thrusters *ω_R_* = *ω_L_*	Nil
Fixed Wing	Heave	90°	90°	Increase or decrease proportionally *ω_R_* = *ω_L_*	Nil
	Roll	90°	90°	Same angular velocity in both thrusters *ω_R_* = *ω_L_*	Aileron input
	Pitch	90°	90°	Same angular velocity in both thrusters *ω_R_* = *ω_L_*	Elevator input
	Yaw	90°	90°	Differential increase in angular velocity *ω_R_* > *ω_L_* or *ω_R_* > *ω_L_*	Rudder input

Here, zero control input leads to no change in angle in the control surface and is abbreviated as “Nil” for control surface deflection. Similarly, 0° motor tilt implies that the motor pod is aligned along the negative heave (Z_B_) axis, and a 90° positive tilt makes it parallel to the surge (X_B_), axis as shown in Figure 2.
